# Method Overview for Discovering ATE1 Substrates and their Arginylation Sites

**DOI:** 10.1002/cbic.202500663

**Published:** 2025-10-28

**Authors:** Richard M. Searfoss, Benjamin A. Garcia, Zongtao Lin

**Affiliations:** ^1^ Department of Biochemistry and Molecular Biophysics Washington University School of Medicine St. Louis MO 63110 USA; ^2^ Department of Medicinal Chemistry Rutgers University Piscataway NJ 08854 USA

**Keywords:** arginylation, ATE1, cDNA screening, proteomics, radiolabeling

## Abstract

Arginylation is a protein modification event in which cellular machinery recognizes a conserved N‐terminal or side‐chain motif and post‐translationally installs an arginine residue to signal a protein for degradation. This modification affects protein function, stability, and half‐life and is essential to proper functions in mammalian systems. Since its discovery in the early 1960s, scientists have struggled to broadly characterize this modification in its canonical function outside of a handful of specific cases. It is known to be an essential cellular mark, as loss of the installation enzyme is embryonically lethal. However, the discovery of the substrates regulated by this mark has been slow and has required some creativity by the scientists who have chased it. Over the course of roughly six decades, the library of substrates has consistently grown through various applications. Here, we seek to summarize all approaches that have been applied to discovering and studying arginylation.

## Graphical abstract

Arginylation is a decades‐old post‐translational modification but is inherently difficult to study. A plurality of methods have been developed and deployed to uncover the biology and substrates of this modification. This review covers the history, major advancements, and substrates discovered over the course of six decades.

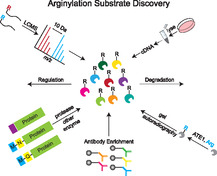

## 1 Introduction

The N‐degron system is a protein degradation pathway where the N‐terminal residue of a protein regulates protein function and half‐life as mediated by degradation signals, or “degrons.”^[^ ^1^ ^]^ Specific enzymes (e.g., E3 ligases) identify these N‐terminal destabilizing residues and post‐translationally modify them whereby ubiquitin conjugating enzymes and complexes, called recognins, recognize these noncanonical modifications and attach polyubiquitin chains to mark the protein for degradation. To date, it has been shown that all 20 amino acids can serve as an N‐degron, and they are regulated by five N‐degron pathways in eukaryotic systems.^[^ ^2^ ^]^ One of the earliest N‐degron systems is the Arg/N‐degron system in which N‐terminal arginine residue was either exposed as part of protein sequence or conjugated by the ATE1 enzyme to N‐terminal aspartic and glutamic acids. Further work revealed that various oxidized cysteine states and deamidated asparagine and glutamine are also substrates of this pathway.^[^ ^3^ ^]^ The Arg/N‐degron has been demonstrated to be a biologically essential pathway, because embryonic and conditional knockout studies have been shown to be lethal in the absence of its function.^[^ ^4^ ^,^ ^5^ ^]^

Although the enzymatic pathway and function of arginylation are understood downstream of the modification, the substrates targeted by this pathway are still largely unknown. Until recently, only a relatively small number of features were known to be arginylated, and some of them lacked undisputable evidence of their validity. However, some substrates have been subjected to thorough investigation, including calreticulin, a‐synuclein, and actin, leading to an increased understanding and interest in arginylation as a field.^[^ ^6^, ^7^, ^8^ ^]^ There are several reasons for the lack of identified substrates of this pathway. First, the current understanding is that the abundance of this modification is low, although there is even evidence seen of the contrary. Given the dynamic range currently observed in traditional proteomics studies, this makes identification without enrichment very challenging. Adding to this, arginylation targets a protein for degradation, and the shorter lifespan of arginylated proteins makes their measurement less likely. Second, it is difficult to distinguish the post‐translational modification from a translational integration of an N‐terminal arginine. Compounding this problem is the use of trypsin as a protease for proteomics experiments, which can be subject to missed cleavages and arginine residues residing at the N‐terminus if proceeded by another arginine or lysine residue. Cleavage in general as a process can be accomplished by numerous proteases in vivo (in cell) and thus is generally unpredictable as a method for finding arginylation substrates. Third, as will be discussed later here, there have been no good enrichment methods to date that are specific to arginylated substrates as compared to proteins containing arginine. As such, the field has developed tools to understand the Arg/N‐degron pathway as system, but has come up short in reproducibly distinguishing the substrates that interact with it.

Since the end result of arginylation is the appearance of an arginine residue as a part of the protein sequence, traditional protein sequencing methods have likely missed arginylation events based on analytical assumptions. Only recently have more unbiased methods been developed to advance the arginylation field and progress into deep discovery of novel arginylation substrates. Given the challenges that are still faced in the discovery of arginylated substrates, this work aims to summarize the main approaches that have historically been used to study arginylation, discover new substrates, recent advances in technology to expand the arginylome, and the future directions of the field. All of these methods have tried to overcome a certain challenge and demonstrate the tenacity of the contributors of the field. Challenges include the ribosome bias, addressed by using ribosome‐free assays, generation of antibodies that are specific to N‐terminal arginylation events that address “contamination” of translationally installed arginine, and using mass spectrometry for higher throughput and specific protein sequencing. This review will compile such methods chronologically, focusing on the advantages and shortcomings in the discovery of arginylation and not the biological context of such events **Table** **1**.

**Table 1:** Substrates of the Arg/N‐degron system and methods used for their discovery.

Substrate	Site(s)	Method
Serum albumin	N‐terminal	Radiolabeling^[^ ^14^ ^]^
*β*‐Melanocyte stimulating hormone	N‐terminal	Radiolabeling^[^ ^19^ ^]^
Angiotensin II	N‐terminal	Radiolabeling^[^ ^19^ ^]^
CALR	E18	Antibody, bottom‐up, top‐down^[^ ^40^ ^,^ ^42^ ^,^ ^44^ ^]^
RGS4/5/6/7/16	C2	cDNA screening^[^ ^31^ ^,^ ^45^ ^]^
SNCA	E46, E83	Antibody/bottom‐up proteomics^[^ ^7^ ^]^
*β*‐Amyloid	N‐terminal	Radiolabeling^[^ ^46^ ^]^
HSPA5 (BiP)	E19	Antibody^[^ ^47^ ^]^
Tropomyosin‐receptor kinase‐fused gene (TFG)	N‐terminal	MetAP cleavage^[^ ^35^ ^]^
BRCA1	D1156	Protease cleavage^[^ ^34^ ^]^
CDC6	D101	Protease cleavage^[^ ^34^ ^]^
PDI	D18	Protease cleavage^[^ ^34^ ^]^
ACTB	D3	Antibody/bottom‐up proteomics^[^ ^8^ ^]^
Actin	E74	Bottom‐up proteomics^[^ ^48^ ^]^
Filamin A	P2151, F2311 and Y2501	Bottom‐up proteomics^[^ ^49^ ^]^
Tubulin	S144, A64, E125, E77	Antibody/bottom‐up proteomics^[^ ^32^ ^,^ ^50^ ^]^
MYH2A	E1169	Bottom‐up proteomics^[^ ^48^ ^]^
MYH2B	E887, E1005, E1012, E1166, E1500	Bottom‐up proteomics^[^ ^48^ ^]^
Alpha‐actinin	D456, D462, D465	Bottom‐up proteomics^[^ ^48^ ^]^
Myosin‐binding protein C	E162	Bottom‐up proteomics^[^ ^48^ ^]^
Troponin T	D63, D72	Bottom‐up proteomics^[^ ^48^ ^]^
Ubiquitin carboxyl terminal hydrolase	D439	Bottom‐up proteomics^[^ ^48^ ^]^
F‐actin‐capping protein	E22	Bottom‐up proteomics^[^ ^48^ ^]^
Creatinine kinase	D326, D335	Bottom‐up proteomics^[^ ^48^ ^]^
ERO1A	E24	Bottom‐up proteomics^[^ ^40^ ^]^
SSBP	E17	Bottom‐up proteomics^[^ ^40^ ^]^
A1AT	E25	Bottom‐up proteomics^[^ ^40^ ^]^
Tau	E3, N644	Bottom‐up proteomics^[^ ^40^ ^]^

## 2 Discovery and Historical Perspective

**Figure 1:**
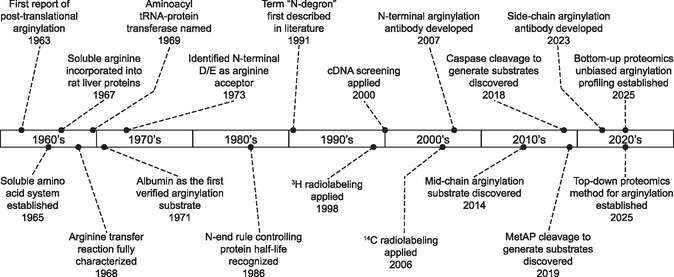
Timeline of major advancements in arginylation discovery and method development.

Arginylation as a PTM is still fairly uncommon in modern research compared with the more abundant ones such as phosphorylation, glycosylation, ubiquitination, and others. However, knowledge of this modification dates back to the early 1960s when it was first discovered that arginine and other residues could be incorporated into proteins in solution and in the absence of ribosomes (**Figure** **1**).^[^ ^9^, ^10^, ^11^ ^]^ Through a number of follow‐up studies in soluble protein fractions from rat liver, it was found that arginine was quite actively incorporated and that it was likely at the protein N‐terminus.^[^ ^12^ ^,^ ^13^ ^]^ The full transfer reaction, rates, and solution requirements were established, and the enzyme was crudely purified and named the “arginyl tRNA‐protein transferase.” Soon after, the N‐terminal installation was validated by arginylation of serum albumin followed by chymotryptic digestion and Edman degradation.^[^ ^14^, ^15^, ^16^, ^17^ ^]^ Continuing in the characterization of this process, it was finally determined that N‐terminal dicarboxylic acid containing residues (aspartic and glutamic acid) were required as an arginine acceptor.^[^ ^18^ ^]^ Even this has been further expanded to show that oxidized cysteine, or deamidated asparagine and glutamine, can also be acceptors. These early efforts were pivotal in establishing the first proof of arginylation as a PTM, as well as demonstrating that the biological process could be reconstituted in a cell‐free environment.

This work continued for decades, though the identification of substrates largely focused on readily available circulatory proteins and hormone peptides, and the role of these modifications was not determined for decades.^[^ ^19^ ^,^ ^20^ ^]^ It was not until studies were conducted on the half‐life of proteins as determined by their N‐terminal residues that revealed the high turnover of N‐terminally arginylated peptides, indicating arginylation as a degradation signal.^[^ ^21^, ^22^, ^23^ ^]^ Culminating this work into a field of its own, the term N‐degron was first coined in 1991 by Alexander Varshavsky when he first saw the need to distinguish the various protein degradation target signals (Figure 1).^[^ ^24^ ^]^ With time, the recognition of the potential roles for degrons in protein degradation, including both N‐ and C‐terminal degrons, quickly grew and garnered more focus from the research community. This expanded in only a few decades to demonstrate the role of arginylation in processes outside of solely protein degradation, such as cytoskeleton regulation and neurodegeneration.^[^ ^7^ ^,^ ^8^ ^]^ As such, the creativity of a select few has pushed this field forward, as new methods were developed de novo to bring arginylation into the limelight.^[^ ^1^ ^,^ ^2^ ^,^ ^25^ ^]^

### 2.1 Radiolabeled Arginine and Autoradiography

**Figure 2:**
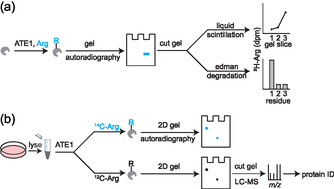
Schemes of protein radiolabeling using heavy arginine. a) Labeling of proteins with ^3^H‐arginine visualized with gel electrophoresis, and confirmed the modification at the N‐terminus with Edman Degradation. b) Labeling of proteins with ^14^C‐arginine or ^12^C‐arginine, where the ^14^C‐arginine labeled protein gel is used as a guide to excise targets from the ^12^C‐arginine gel before being subjected to mass spectrometry.

As more information was revealed about the N‐degron system through targeted studies, the need for the development of broader techniques for evaluating arginylation arose. One such technique was using radioactive arginine and autoradiographic imaging in which the installation of labeled arginine onto proteins could be directly visualized by their emitted radiation (**Figure** **2**). The primary benefit of this method is in its sensitivity. Even compared with the modern sensitivity of mass spectrometry, any trace of installed radiolabeled arginine can be detected across a wide dynamic range, and this is beneficial to studying arginylation of proteins given the natural low abundance of the mark. An early study utilizing this approach used [^3^H]arginine to measure the incorporation of arginine in solution, and importantly, in the absence of translation machinery.^[^ ^26^ ^]^ It was shown that incubating rat brain homogenate with [^3^H]arginine resulted in incorporation into endogenous proteins only in the presence of ATP. Further, arginine was primarily installed onto the protein N‐terminus as the radioactivity was lost following a single round of Edman degradation, though the exact proteins that were arginylated here could not be determined. This method was then used to arginylate artificially oxidized BSA and RNaseA, which were only arginylated in their oxidized form and not in their native forms. This linked oxidation damage to the arginylation degradation pathway, as well as noncarboxylic acid containing N‐terminal residues, as RNaseA contained an N‐terminal lysine which was arginylated. This study inspired numerous others using autoradiography.^[^ ^7^ ^,^ ^27^, ^28^, ^29^ ^]^

This approach was further applied and coupled with mass spectrometry to definitively determine the proteins that were being arginylated as identified by earlier studies.^[^ ^30^ ^]^ Using similar rat brain homogenate and now coupled ^12^C‐Arg and ^14^C‐Arg, ^14^C‐Arg labeled bands identified in 2D gel were excised from the ^12^C‐Arg labeled gel, and these protein spots were extracted and subjected to mass spectrometry. Here, they were able to identify six arginylated peptides belonging to proteins that are proteolytically processed to remove an endoplasmic reticulum signal peptide, revealing N‐terminal amino acid substrates belonging to the Arg/N‐degron.

Until higher throughput and broadly compatible methods were developed for substrate discovery, autoradiography remained a widely popular approach for finding, monitoring, and validating arginylation events due to its high sensitivity and unambiguity. However, it still presented its challenges. In general, it was a low throughput approach requiring extensive experimentation with ultimately low yield. When finally coupled to mass spectrometry, it had a reliance on perfect reproducibility between gels and exact extraction of unlabeled bands based on the location of radiolabeled bands, and there remains the chance that many substrates were still missed. It is also possible that autoradiography was able to detect labeled substrates with its high sensitivity that still fall below the limit of detection of the mass spectrometer. Still, this approach provided important insights into the apparent diversity of arginylated substrates present in the matrices it was applied to and still demonstrated clear evidence of the arginylation process.

### 2.2 cDNA Screening

**Figure 3:**
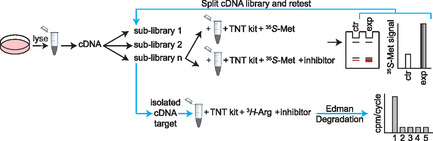
cDNA library screening for the discovery of arginylation substrates. Sublibraries of 50 cDNAs were consecutively expressed in the presence or absence of Arg/N‐degron inhibitor to identify libraries with increasing concentration in the presence of inhibitor. Libraries with increasing protein concentration were consecutively split into smaller libraries (25 + 25, 12 + 12, 6 + 6, etc.) until a single cDNA was isolated and determined to be increasing in concentration due to being an arginylation substrate. The isolated cDNA was then confirmed to be arginylated and at the N‐terminus by the loss in signal through a single round of Edman Degradation.

A different study attempting to broadly identify substrates of arginylation‐mediated degradation used cDNA screening in several applications (**Figure** **3**).^[^ ^31^ ^]^ In cDNA screening, mRNA is isolated from a matrix of interest, converted to cDNA, and inserted into plasmids that are then transformed into *E. coli* to generate a cDNA library. This library can then be split into smaller pools to determine which pools contain active proteins in a given process or substrates of a pathway by monitoring activity, and then pools can be repeatedly divided until the protein of interest is identified. Since the goal of this study was to determine the substrates of the arginylation pathway, an in vitro transcription/translation system was used to monitor protein translation and subsequent degradation by proteins containing radiolabeled [^35^S] methionine in two different approaches.

First, a fusion protein bearing an N‐terminal ubiquitin tag, a residue of interest, and a portion of the viral polyprotein nsP4 was created in cDNA pools and produced in rabbit reticulocyte lysate using the in vitro transcription‐translation‐degradation system. Deubiquitylating enzymes in solution would cleave the N‐terminal ubiquitin, leaving an open destabilizing residue. After protein translation peaked, protein degradation was monitored over time in the presence or absence of dipeptide inhibitors of the N‐degron system. They demonstrated that the R‐nsP4 was readily degraded after peak translation and that degradation could be inhibited by the addition of the Arg‐*β*‐Ala inhibitor, indicating R as a degradation signal and validating this system as suitable for the discovery of arginylated substrates.

To broadly search for substrates of the Arg/N‐degron system, pools of cDNA generated from mouse brain RNA were added to the same transcription‐translation‐degradation system in reticulocyte lysate. Here, expression was carried out in the presence or absence of Arg‐*β*‐Ala and Trp‐Ala dipeptide degradation inhibitors, looking for proteins that increased in concentration in the presence of inhibitors but not in the absence of inhibitors through an increase in [^35^S]methionine signal. This resulted in the identification of seven total cDNAs with altered expression, including the full‐length RGS4 as a putative substrate of the Arg/N‐degron system. This finding was validated through [^3^H]arginine radiolabeling and Edman degradation where [^3^H]arginine was found to be incorporated onto the N‐terminal cysteine of RGS4 following Met cleavage by MetAPs. Taken together, they systematically verified the use of arginine as a destabilizing N‐degron and demonstrated its use on a substrate newly identified through cDNA screening.

This approach has the benefit of being adapted to any protein substrate or biological matrix of interest and was able to identify a previously unknown substrate of the Arg/N‐degron system. Further, it provides the benefit of only realistically needing a single copy of a transcribed gene to be tested within the sample, meaning that even an extremely low basal translation of a protein can be included in the assay. However, it is time and experimentally intensive for what resulted in a low yield. Given that this was only tested on a single tissue source and on actively transcribed genes, it was not a comprehensive assessment of the heterogeneity of arginylation substrates. Further, the targets could have been removed following transformation by degradation of plasmids by *E. coli* host machinery.

### 2.3 Antibody Enrichment for N‐Terminal Arginylation

**Figure 4:**
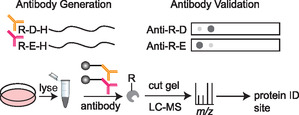
Application of antibody enrichment for N‐terminal arginylation. Antibodies were raised against N‐terminally arginylated peptides and had confirmed specificity with dot blots that positively bound to N‐terminally arginylated peptides and did not bind to peptides of similar sequence but with no arginylation. Antibodies were then coupled with beads and applied to cell lysate to enrich arginylated proteins that were identified by LCMS analysis.

As technology progressed, and the relative throughput of experiments increased allowing more intensive analyses to be established, the coupling of enrichment via antibody pulldown and identification through gel and mass spectrometry emerged (**Figure** **4**).^[^ ^32^ ^]^ The hope here was that by using specific enough antibodies, arginylation substrates could be enriched out of a sample and identified without interference from unarginylated proteins. Since it was already established that N‐terminal aspartic and glutamic acid residues were the primary targets, antibodies were raised against peptides containing RD‐ and RE‐motifs at the peptide N‐terminus and coupled to affinity beads for immunoaffinity chromatography. Here, a variety of mouse tissues were processed to cast as wide a net as possible through the mouse proteome. After bottom‐up proteomics mass spectrometry analysis, and strict filtering of matching peptides with one validated with a synthetic peptide, a total of 43 verified peptide substrates were able to be identified, including actin which was previously known to be arginylated, thus further validating this approach. This was by far the most comprehensive study to date and identified the largest pool of arginylated substrates but also provided some confounding results. Here, they provided evidence of arginylation on peptides containing almost every amino acid at the N‐terminus, including native (nondeamidated) asparagine and glutamine, as well as native (non‐oxidized) cysteine. Further, given the location in the full protein sequence that some of these sites were identified as away from the N‐terminus but still on the N‐terminus of the peptide, this provided the possibility of sidechain arginylation if the protein was not proteolytically processed prior to arginylation. The team applied this methodology to continue finding substrates. Reprocessing of the data by further experimentation validated the presence of arginylation occurring on Asp and Glu sidechains in the middle of the polypeptide chain.^[^ ^28^ ^,^ ^33^ ^]^ While this study made strides in discovering new substrates as well as new N‐end rules for arginylation, it still did not address the possible ribosome bias to differentiate truly arginylated substrates compared with translationally arginylated ones. Further, all the new substrates reported were not validated with synthetic peptides, and did not provide clear enough evidence of arginylation in the fragment spectra. Given that these experiments were conducted in the early days of proteomics when instruments were still of low resolution, and the discovery relied exclusively on searching algorithms, while promising, these results still required further analysis for validation.

### 2.4 Arginylation of Protein Cleavage Products

**Figure 5:**
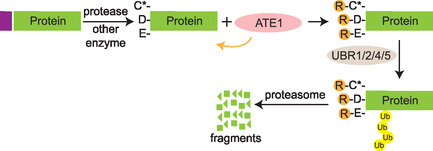
Schematic depicting the generation of substrates through endoproteolytic cleavage for arginylation and subsequent protein degradation. Proteins are first cleaved by a protease to reveal an N‐terminal D/E or C requiring oxidation, which can then be recognized and arginylated by ATE1 and passed through the UBR‐mediated ubiquitination and proteasomal degradation pathway.

Another study sought to probe the interaction between endoproteolytic cleavage of proteins during co‐ and post‐translational processing and the Arg/N‐degron pathway (**Figure** **5**).^[^ ^34^ ^]^ Endoproteolytic cleavage, in which a protein is cleaved midchain 3 or more residues internal from either terminus, is an important mechanism for protein processing to remove signaling peptides, activate proteins, respond to stress, and basal turnover. However, the products of these processes were only understood to be degraded and recycled, though the mechanism of how was unclear. In vivo study showed that the caspase cleavage sequence was required for cleavage of CDC6, which resulted in an open ASP101 that was then arginylated in ATE1^+/+^ cells and was degraded by the ubiquitin‐proteasome system in the absence of the proteasome inhibitor MG132. RD^101^‐CDC6 can also be accumulated in cells treated with MG132 as well as autophagosome inhibitor hydroxychloroquine. When not treated with hydroxychloroquine, RD^101^‐CDC6 were observed to localize with the autophagosome adapter protein p62, indicating that in the absence of proteasomal activity, the autophagosome can substitute for protein degradation and turnover within the Arg/N‐degron system. A similar phenomenon was observed with the caspase‐cleaved and arginylated RD^1156^‐BRCA1, where the cleavage product accumulated under blockage of both the ubiquitin‐proteasome system and autophagosome, but was degraded under only the active autophagosome. Further, an increase in arginylation of known substrates could be induced by HSP90 inhibition which results in high concentrations of misfolded proteins. Although only four known targets were evaluated, this postulates that other misfolded proteins are likely arginylated to process this cellular stress.

This study showed that proteolytically processed proteins, in addition to native proteins, can be substrates of the Arg/N‐degron pathway, and that this pathway interacts with both major protein degradation machineries under stress conditions. Although the focus was less on broad substrate discovery, it did provide a possible reason for why relatively low numbers of substrates have been documented thus far in the arginylation field, as well as new processes for generating substrates of the Arg/N‐degron system. Such a method relies on a priori knowledge of proteolytic cleavage of proteins and thus could only serve as a validation approach rather than discovery tool. Regardless, the theoretical substrate pool significantly increased when considering the translational and proteolytic processes available to generate substrates. However, by focusing on only known targets and not trying to more broadly discover arginylated proteins after inducing stress, the broader interactions between these systems still requires attention.

### 2.5 N‐Terminal Protein Processing for Arginylation Substrate Generation

**Figure 6:**
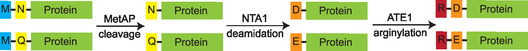
Schematic of the generation of arginylation substrates from proteins containing N‐terminal methionine. A protein is cleaved by MetAP, and/or an N‐terminal asparagine or glutamine that is deamidated to ATE1 compatible aspartic and glutamic acid, respectively.

Besides caspase cleavage of proteins as a co‐translational protein processing step, cleavage by exoproteolytic MetAPs also routinely occurs to remove N‐terminal methionine when followed by small, uncharged amino acids. However, it has been found that this process occurs in a subset of proteins containing asparagine and glutamine residues following methionine, which are easily targeted by deamidating enzymes that convert them into aspartic and glutamic acids and are targetable by the Arg/N‐degron pathway. A study verified Met cleavage when followed by N/Q using synthetic peptides harboring these motifs (**Figure** **6**).^[^ ^35^ ^]^ A reporter fusion protein bearing an N‐terminal Ac‐MN motif was degraded in yeast by the Ac/N‐degron system, and the same reporter bearing N‐terminal MN motif was degraded by the Arg/N‐degron system following MetAP cleavage and NTAN1 deamidation of N to D which then underwent ATE1‐mediated arginylation and subsequent degradation. The same phenomenon was observed in fusions bearing N‐terminal Ac‐MQ and MQ motifs. With clear evidence of the interaction of the MetAP pathway with the Arg/N‐degron pathway, they sought to screen the yeast proteome for other substrates. In their analysis, they applied a yeast‐2‐hybrid (Y2H) screen where two proteins of interest, a bait and a prey, are fused to transcription factors, and if they interact, then a reporter gene is activated signaling their interaction. Here, a UBR1 construct containing the N‐recognin domain was used as bait against various cDNA libraries used as prey. In two systems, they identified the human tropomyosin‐receptor kinase‐fused gene (TFG) with a resulting N‐terminal MN motif, and the *S. cerevisiae* nucleotide excision repair protein Rad16 with a resulting N‐terminal MQ. Both substrates were degraded following MetAP cleavage and deamidation prior to ATE1‐mediated arginylation, and both were stabilized following ATE1 knockout. This study verified the MetAP processing of native proteins containing destabilizing residues following methionine that generate substrates of the Arg/N‐degron pathway.

Using this system had the advantage of using the UBR1 plasmid in UBR1 KO cells because this is the only Arg N‐recognin within yeast, so it was specific to the arginylation substrates. However, it had similar challenges to using the previously discussed cDNA screening. Further, considering MN/MQ‐starting proteins encompass 9% of the encoded yeast genome, or roughly 600 proteins, this approach also only identified one new substrate, Rad16, which started with an MQ N‐terminus.^[^ ^36^ ^,^ ^37^ ^]^ The yield from this approach was low given the possible substrates.

### 2.6 Antibody‐Based Identification of Sidechain Arginylation

**Figure 7:**
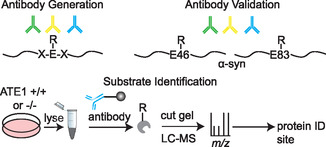
Application of antibody enrichment for side‐chain arginylation. Compared with the N‐terminal arginylation antibodies, here antibodies were generated against synthetic peptides containing glutamic acid residues in the middle of the polypeptide chain that were arginylated on the carboxylic acid side chain. These antibodies were validated against *α*‐synuclein side‐chain arginylated peptides before being applied to immunoprecipitation and mass spectrometry.

A number of studies postulated about, and began to provide evidence of, the presence of sidechain arginylation, or the addition of arginine to the functional group of aspartic and glutamic acid linked through the side chain carboxyl and the N‐terminal amine of the added arginine.^[^ ^32^ ^]^ However, the use of antibodies for enrichment of arginylated substrates followed by mass spectrometry identification still resulted in relatively low yield compared with the theoretical abundance of arginylation supported by high incorporation of radio‐labeled arginine. Further, no antibody had been developed to specifically enrich for and validate side‐chain arginylated substrates in a discovery setting. This led to the development of a “pan‐arginylation” antibody for immunoaffinity enrichment (**Figure** **7**).^[^ ^38^ ^]^ The main difference between this approach and the first arginylation antibody was in the antibody design. Here, the authors instead raised antibodies against a synthetic glutamic acid‐sidechain arginylated peptide library, with the theory that it would be able to enrich both aspartic and glutamic acid sidechain arginylated proteins, as well as potentially enriching N‐terminally arginylated peptides on the canonical D/E. By doing this against an entire library of random sequence peptides, it should be more specific to the D/E + R motif and depend less on the surrounding sequence. With three successful antibodies generated, cell lysates were subjected to immunoaffinity chromatography to enrich sidechain arginylated proteins, and following mass spectrometry analysis, they were able to identify 17 sidechain arginylation sites with high confidence, and several other sites that were confident but did not pass strict filtering criteria for various reasons. As such, this was another successful study in the use of antibodies to probe the arginylome, because they found not only new substrates of arginylation but also identified previously known substrates, partially validating the basis of these experiments. However, as noted by the authors, this is still likely an underestimation of the true arginylation presence given that each antibody likely has slightly different substrate specificities and compared with the arginylation levels seen with the previously discussed radiolabeling. Also, no experimental validation was performed on the listed sites seen here, so there is still some doubt on the credibility and validity of what was determined to be arginylated. Still, this study was another major step forward in expanding the possible library of substrates of the Arg/N‐degron system by now including sidechain arginylation as a target.

### 2.7 Isotopically Labeled ATE1‐Based Arginylation Profiling

**Figure 8:**
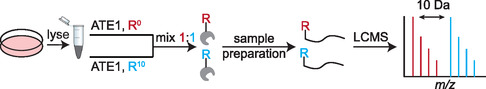
General overview of the bottom‐up proteomics workflow. A protein, cell, or tissue of interest is split into two aliquots, incubated with ATE1 and isotopically labeled arginine and then recombined. Following typical bottom‐up proteomics sample processing and LCMS analysis, arginylated peptides can be identified by the presence of co‐eluting doublets of isotopic peaks 10 Da apart, corresponding to the heavy and light arginine.

Bottom‐up proteomics is a ubiquitous technology that is routinely applied to in‐depth discovery analyses in the modern age of research. Even in its infancy, this approach was used to try to discover new arginylation sites, with some previously mentioned here.^[^ ^30^ ^,^ ^32^ ^,^ ^39^ ^]^ However, they all lacked the depth of coverage of the entire proteome due to the requirement of enrichment prior to analysis, as well as a way to distinguish substrates in an unbiased manner, as a number of induced modifications or combinations of residues closely match the mass shift of arginine.^[^ ^32^ ^]^ Recently, a reinvention of prior approaches was developed to address previous shortcomings termed ATE1‐based arginylation profiling (ABAP).^[^ ^40^ ^]^ Here, instead of using radiolabeled arginine, which is not compatible with mass spectrometry analysis due to safety reasons, ^13^C^15^N heavy‐labeled arginine was applied instead. Using a similar in vitro ATE1 arginylation assay supplemented with either ^12^C^14^N (light) or ^13^C^15^N (heavy) arginine, labeled mixtures were then combined, deeply fractionated offline, and subject to bottom‐up proteomics mass spectrometry analysis (**Figure** **8**). This approach provided the benefit of site validation because the heavy labeled arginine could only be integrated by the ATE1 assay, and MS1 signal would show co‐eluting labeled peptides separated by a 10 Da mass shift (the mass difference of the heavy and light arginine). At the fragment level, the same spectra would be created for *y*‐ions generated from the C‐terminus, and the same spectra offset by 10 Da would be created for *b*‐ions from the N‐terminus, providing a second piece of validation within the same experiment. When applied to many different complex matrices, and validated with synthetic peptides, hundreds of sites were identified, the largest increase in new sites to date. As an assay, the throughput is on‐par with other previously mentioned methods when considering cell culture or tissue procurement, assay‐based labeling, fractionation, and analysis. Given the requirement of mass spectrometry analysis and here, a newly development software specific to searching for the MS1 pairs, this is still not a broadly applicable method to the entire arginylation field. It also required high levels of fractionation, extensive instrument time, and no enrichment of sites, so the dynamic range of what was able to be discovered is still a challenge. Regardless, it is probably the best advancement to the arginylation field since the discovery of this modification.^[^ ^41^ ^]^

### 2.8 Top‐Down Proteomics

**Figure 9:**
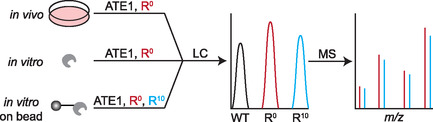
Top‐down proteomics approaches to discovery and validation of arginylated substrates. Arginylation was carried out in vivo by co‐expressing CALR and ATE1, in vitro by applying the same arginylation assay as bottom‐up proteomics, and in vitro but on‐bead during protein pull down assays, also using isotopically labeled arginine. Proteins are then quantified and sequenced with LCMS.

Although the depth and throughput compared to bottom‐up proteomics approaches are still lacking, top‐down proteomics now rivals early bottom‐up proteomics experiments. This approach provides the primary benefit of identifying the full sequence of the protein, including distinguishing between sequence isoforms, and PTM‐presence at the intact protein level, sometimes even with specific site localization. However, until recently, no top‐down proteomics experiments have ever been performed analyzing intact arginylated proteoforms for validation and discovery. Given the difficulties associated with top‐down proteomics, including dynamic range issues and signal spreading due to multiple charge states and isotopes, it is not surprising that it was not employed in this field. To demonstrate if it was a possible tool to study arginylation, calreticulin was chosen as a use case due to its previously demonstrated arginylation efficiency and utility as a standard.^[^ ^6^ ^,^ ^30^ ^,^ ^42^ ^,^ ^43^ ^]^ Here, top‐down proteomics was shown to be able to differentiate between unmodified and arginylated calreticulin, measure its arginylation in vitro and in vivo, quantify the levels of arginylation, and utilize isotopically labeled arginine for substrate validation (**Figure** **9**).^[^ ^44^ ^]^ This was the first use case of evaluating intact protein substrates of the Arg/N‐degron system. It has been further demonstrated by measuring the in vivo arginylation and further leucinylation of ERO1A and SSBP1 fusion peptides, supporting the applicability of top‐down proteomics as a tool.^[^ ^40^ ^]^ By measuring arginylation at the intact level, in future studies, interactions between arginylation and other post‐translational modifications can be explored. At present, this has been shown as a useful tool for individual proteins, but for both arginylation and proteomics studies broadly, it still has the drawbacks of being low throughput, low sensitivity, and difficult to generalize because of the different chemical natures of protein classes.

## 3 Summary and Outlook

Advancements in the arginylation field have appeared to be slow, occurring over the span of 6 decades, but they have kept pace with the general advancements in science and technology, often being applied to arginylation by a few labs as techniques gained popularity and traction. The most recent advancements in bottom‐up and top‐down proteomics utilizing heavy and light labeled arginine as a self‐validating substrate may be the largest step forward yet, but even here, the approaches are still a few steps behind the rest of the mass spectrometry field. First, many in the field are focusing their efforts on advancing data‐independent acquisition (DIA), which differs from the earlier bottom‐up proteomics experiments where a single peptide target was analyzed at a time. In DIA, multiple peptides that are close in mass within a given mass range are analyzed at the same time, providing the benefit of increased depth of coverage by spending less time on individual targets, as well as analyzing even lower‐abundant species that are near the limit of detection of the instrument because they are co‐isolated with abundant features. This could bring great promise to finding new arginylated substrates that were missed due to their low abundance in the previous studies, but it comes at the cost of increased computational and statistical needs. In general, DIA software still lacks its ability to do de novo sequencing of peptides because their true strength is in referencing spectral libraries. When applied to arginylation and the requirement of true discovery‐based proteomics, they still fall behind in their utility. With time, the improvements in mass spectrometry sensitivity and resolution, coupled with ion‐mobility separation, should greatly benefit this field. In the case of top‐down proteomics, throughput is still a major challenge, but the technique is now there to apply to more complex mixtures and validate previously found substrates in bottom‐up studies, with the added possibility of finding specific proteoforms that are regulated and/or degraded by arginylation. Other approaches that will greatly enhance the study of arginylation include N‐terminal enrichment approaches that could be targeted to arginine, as discussed in a separate note.^[^ ^41^ ^]^

In closing, the arginylation post‐translational modification has garnered low but consistent attention since its discovery in the 1960s. Although progress has been slow, major leaps have been made in understanding this essential pathway, as well as other N‐degrons. The credit for this work can be given to a select few labs, who saw the importance of treading into the unknown to chase after such a challenging mark. In comparison, phosphorylation is a widespread, routinely analyzed post‐translational modification mainly due to its biological nature regulating protein activity and its relative abundance in the proteome. Similar arguments can be applied to ubiquitination, acetylation, glycosylation (although there are many complexities here as well), and now many ‐acyl marks. One could claim that these are “easier” to study since they are easily distinguishable from the protein itself when compared to arginine. As such, the tools to study these other PTM's are readily available, commercialized in kits and antibodies, and widely accepted across the scientific community. In the case of arginylation, the widespread utility of the methods highlighted in this review still lack, but they hold the ability to shine light on the ever‐growing roles of arginylation.

## Conflict of Interest

The authors declare no conflict of interest.
